# Gambling as social practice: a complementary approach for reducing harm?

**DOI:** 10.1186/s12954-019-0342-2

**Published:** 2019-12-05

**Authors:** Ross Gordon, Gerda Reith

**Affiliations:** 1 School of Advertising, Marketing and Public Relations, QUT Business School, Queensland University of Technology, Brisbane, Australia; 2 School of Social and Political Sciences, University of Glasgow, Glasgow, UK

## Abstract

**Background:**

Gambling is now a well-recognised public health issue and forms the focus of extensive harm reduction initiatives. Recent developments in policy, practice and technology, such relaxation of regulations, the increasing influence of global gambling corporations, and the development of devices such as mobile phone apps and fixed odds betting terminals (FOBTs) mean that the landscape is a complex, dynamic, and fast moving one. Gambling is now practiced using new technologies, in various spaces and places, and features in a range of social surroundings. Therefore, research is needed to inform appropriate gambling harm reduction strategies that can respond to this complex domain. Yet, research and policy approaches to the reduction of gambling harm are predominantly framed through psychological and economic models of individual behaviour, addiction, and ‘rational’ action. This is beginning to change, with a growing corpus of socio-cultural approaches to gambling research now emerging.

**Method:**

In this article, we argue the case of recognising gambling as a social practice, the performance of which draws upon multiple elements such as technology and materials, spaces and places, language and discourse, and structures and agency. We call for a practice theory approach to gambling research that joins efforts to move beyond individual gamblers and their behaviour, to also acknowledge the interaction of multiple elements shaping gambling practices. To achieve this, we suggest that research methods such as visual ethnography can be helpful.

**Conclusion:**

We set out how a social practice perspective to gambling research can generate different insights and help inform more nuanced and appropriate gambling harm reduction initiatives.

## Introduction

There is growing consensus that gambling is a major public health issue [1]. Health-related gambling harms include headaches and nausea, stress, anxiety, and depression [2]. Social harms on individuals, families, and communities include financial hardship, family breakdown, reduced productivity at work or study, and criminal activity [3, 4]. The social costs associated with gambling are considerable. In this article, we consider the case of gambling harms in Australia and the UK—two countries with a colonial history, shared threads of cultural heritage, English as the de facto language, and legal systems based on common law. Although exact figures can be difficult to identify, gambling-related harms have been estimated to cost between $4.7 billion and $8.4 billion per annum in Australia in 2010 [5], whilst a 2016 study estimated that gambling harm costs up to £1.2 billion per year in the UK [6]. Research has suggested that it is not only pathological or problem gamblers that warrant attention, but also low- and moderate-risk gamblers. Indeed, because of their far greater numbers in the population, the total burden of gambling-related harm from low- and moderate-risk gamblers is higher. For example, a study in Victoria, Australia estimated the total burden of gambling-related harm (health, emotional, financial, performance at work or study, relationship, and neglect of family and friends) to be 50% among low-risk and 34% among moderate-risk gamblers [4], and identified that their potential to transition to more problematic gambling is also of concern.

Researchers have also identified that the gambling environment is dynamic and fast changing [7, 8]. In recent years, new products, services, and technologies such as mobile smartphone sports betting apps, electronic games machines (EGMs), and fixed odds betting terminals (FOBTs) have emerged in Australia and the UK [9]. Furthermore, gambling is heavily marketed by the industry [10, 11], and the policy and regulatory environment has become increasingly liberalised in countries both the UK and Australia [1, 12]. Yet much extant gambling research focuses on pathological and problem gamblers [13]. Furthermore, whilst there is a wealth of gambling research focusing on individual behaviour, addiction, and cognitive impairment, there is a smaller but nascent corpus of knowledge considering the role of the wider socio-cultural, regulatory, and commercial environment that shapes and influences such behaviour [14, 15]. As a consequence, harm reduction strategies could benefit from a broadening of perspectives to acknowledge both individual *and* socio-cultural influences on gambling-related harm. This would help inform interventions that not only seek to correct gamblers’ cognitive deficits, increase their personal awareness of risk, or encourage self-limiting ‘responsible’ behaviours [16], but also address socio-cultural and structural factors such as social norms, spaces and places, marketing, and policy and regulation, of gambling, and which can influence gambling behaviours. In this paper, we argue that to produce more effective and comprehensive ways of reducing gambling harm, the broader social, cultural, and regulatory environment in which gambling behaviour is produced should be acknowledged. Therefore, to help inform gambling harm reduction strategies, interdisciplinary research is needed to understand the complex psychological, social, cultural, and structural influences on gambling practices [17–19].

Responding to the calls for broader socio-cultural perspectives in gambling research [20, 21] this article introduces ideas from practice theory into the area. Recognising the contributions that social practice perspectives have made in other areas of social research and harm reduction policy [22], we argue that framing gambling as a social practice can make discernible contributions to the field. As we explain, practice theory offers a productive lens for ‘flipping the iceberg’ and recognising and understanding the embodied, cognitive, social, and spatial dimensions of social practices of gambling to help generate new insights regarding related risk and harms. Such insights can help inform gambling harm reduction strategies to protect individuals and communities. Our aim is to encourage gambling researchers to engage with the idea of gambling as a social practice and so to contribute to the development of a broader, interdisciplinary research field.

The remainder of this article is set out as follows. First, we appraise the existing gambling research landscape, and then introduce, define, and explain practice theory. Next, we outline how practice theory has been applied in other areas of social research and harm reduction policy and identify the utility of a gambling as social practice perspective. The subsequent section considers what a social practice agenda in gambling research may look like, and identifies some of the research questions, topics, contexts, and methodologies that may be used to carry out such research. We then finish the article with some conclusions for gambling research, policy, and practice oriented towards harm reduction.

## The nature of gambling research and the changing gambling environment

Scholars coming from public health, social, cultural, political, and geographic perspectives have argued that gambling research would benefit from an expansion beyond psychology of individual behaviour perspectives [17, 23, 24]. Much of the existing corpus of gambling research tends to focus on the ‘addictive’ psychology and personality of gamblers, thereby locating the cause of gambling-related harm in individual gamblers [25–27]. Heretofore, there has been less attention paid to the social, political, cultural, and structural environment that shapes gambling practices, albeit work from these perspectives is becoming increasingly prominent [28]. The contemporary gambling research literature is also now a somewhat contested space. Critical scholars have drawn attention to the neoliberal infused political economy of gambling, examining how the gambling industry and policymakers have shaped the global gambling environment through a process of globalisation, liberalisation, marketisation, and exploitation of the poor [12, 17]. However, scholars working from a normative perspective submit that such critique is politicised, ideological, polemical, and often lacking in empirical support, and argue that gambling is a legitimate global industry and consumer behaviour [29]. An advantage of a social practice theory perspective to gambling research is that it potentially offers a way to account for ideas aligned with the critical as well as normative perspectives through consideration of both structure (e.g. how political economy, policies, institutions, norms, rules, expectations, or physical environments shape gambling practices) and agency (the ability for an entity to act and cause an effect e.g. how, where and why humans perform gambling practices).

We argue that foregrounding socio-cultural as well as individual factors that influence gambling is important given the dynamism and increasing complexity of the gambling landscape, particularly within jurisdictions that have been subject to a period of liberalisation and deregulations in recent years [15]. Over the past 30 years, governments have legalised lotteries, casinos, sports betting, and machine-based gambling, dramatically increasing availability and opportunities to gamble, generating considerable state tax revenue, increasing industry profit, and more firmly establishing gambling as a social norm [15, 30]. Given a more relaxed policy and regulatory environment, it is no surprise that the marketing of gambling has been a prominent point of discussion in the literature [31]. Researchers have identified how gambling is now heavily marketed across a broad range of channels such as TV advertising, sponsorship, branding, and social media [10, 11]. The marketing of gambling also utilises appeals to socio-cultural constructs such as rituals, mateship, winning and success, social status, thrill and adventure, hedonism, and sexuality [32]. As we explain in this article, these social constructs lend themselves well to a practice theory framework given its focus on social practices and elements of practice such as norms, rituals, and discourse.

Technological advances have also changed the gambling landscape [33]. Fixed odds betting terminals, a form of electronic gaming machine that allows gamblers to bet on the outcomes of games such as roulette and blackjack which have fixed odds and with the theoretical percentage return to gamblers displayed on the machines, were introduced in the UK in 2001. They have been described in the UK press as the ‘crack cocaine’ of gambling due to their supposedly addictive nature, and it is argued that they are highly concentrated in lower-income communities [34]. Automated gambling products that feature highly engaging game characteristics such as rich graphics and event-based sound effects, and in game information such as statistics, history, and betting options, are now a common feature in casinos [33]. It is argued that such technologies are designed to encourage excessive gambling, or what has been described by Schüll [35] as ‘addiction by design’. Technological advancement of EGMs has also led to the development of some with embedded skill elements.

The introduction of smartphone technologies has generated a dramatic development in the availability, functionality, and increasing use of online sports betting through mobile phone apps [36]. Mobile phone sports betting apps enable people to bet anywhere, anytime, and on anything. Research suggests that sports betting apps can be a prominent feature of social practices of gambling, and socialisation rituals among young people in Australia [20]. They attribute this popularity to their ease of use and their ability to be accessed alone or whilst with friends in a range of venues such as at home, at a sports fixture, or in the pub. Newer innovations such as bet-in-play features have further changed the dynamics of how, when, and why people bet on sports [37]. Research suggests that use of sports betting apps and Internet gambling in general appears to be integrated into everyday living activities and often in a social manner with friends or family [38, 39]. This suggests that sports betting is also a socio-cultural as well as an individual psychological phenomenon [20]. Yet, there is a paucity of research that considers practices of sports betting using mobile phone apps [40].

Furthermore, the spaces and places in which gambling can occur are varied. As well as taking place in traditional venues such as casinos, bookmakers, and betting lounges in pubs—gambling can take place at home, on the bus, at a game, or even on the beach [21]. In an increasingly mobile and social landscape, gamblers are being increasingly connected to new forms of technology, to social networks, and to flows of information that may influence their experience of gambling, and their behaviours, in new and unforeseen ways. We argue that each aspect of this dynamic new environment can be understood through the lens of practice theory. As we explain, practice theory offers a useful perspective for gambling research given its focus on various elements of social practice such as materiality, sociality, and discourse.

## On practice theory

Across the social sciences, theories of everyday social practice have emerged, building upon ideas from scholars such as Bourdieu [41], Giddens [42], de Certeau [43], and Ortner [44]. Since the late 1990s, the work of social practice theorists such as Schatzki [45], Reckwitz [46], and Shove [47] has gained considerable attention. Such work focuses on the dynamics between structure (e.g. policies, institutions, norms, rules, expectations, physical environments) and agency (the ability for an entity whether humans, animals or objects to act and cause an effect), and how various elements in the social world and relations between these shape social practices and related outcomes. Practice theory refers to a broad paradigm of theoretical and methodological socio-material approaches to understanding everyday social practices using a socio-cultural lens [46, 48, 49]. Practice theory provides a dialectic and relational framework for understanding mutual interactions between actors (any person or object that has agency) and the contexts and structures in which they operate. Andreas Reckwitz [46] defines social practices as ‘a routinized way in which bodies are moved, objects are handled, subjects are treated, things are described and the world is understood’ (p. 250). Therefore, social practices refer to everyday or regular practices or habits such as consumption of food [50], using energy in the home [51], or gambling [20], and the way that these are typically and habitually performed in society [48].

Practice theory is now widely used as an approach across several social science disciplines including philosophy [46, 48], sociology [51, 52], anthropology [53], cultural studies [50], geography [51], and marketing and consumer research [54]. Practice theory considers humans as actors, who use their agency within the structures shaping various social practices [46]. Therefore, practice theory treats the abstract concept of social practices as the key unit of analysis. Although various actors and entities interact and overlap when practices occur, the primary focus in this approach is on the practices themselves and not simply on the individual human performers of a practice [55]. People therefore become ‘carriers’ of practices. The benefit of a practice theory approach arises from recognising the multifaceted and situated realities of the everyday ‘doing’ of things. Applied to gambling research, it has the potential to move beyond approaches to that limit their focus to single aspects of human cognition and/or behaviour. At the same time, practice theory offers a new way for thinking about how complex, embedded behaviours social practices such as gambling, can become habitual and routinised within different social contexts. Reckwitz [46] theorises that practice is consisted of different elements such as bodily and mental activities, use of materials, knowledge, language and discourse, norms, social structures, spaces and places, power, and individual/group agency, that are all utilised to routinely perform the practice in question.

The body and how it performs is a core element of practice [46]. Practices are routinised bodily activities, they consist of related behavioural acts that involve movements of the body. Our performance of social practices becomes embodied and we learn how to use our bodies to perform various practices through perception, learning, cultural acquisition through the senses and so on. This embodied knowledge of how to use the body to perform a practice become habitual, part of our skill set, and shape our dispositions [41]. Take for example, the act of placing a bet on a horse race. A gambler may lift up and open a race guide with their hands, read the guide with their eyes, walk to a betting shop using their legs, fill in a betting slip using their hands, and watch the race using their eyes—here various movements of the body are required to perform the practice of betting on a horse race. Another element of practice is the sets of mental activities used to perform them, and which require understanding of the world, desires to do something and associated emotional and affective responses and knowing how to do something. Taking the same example of betting on a horse race, this demands a desire to place a bet, knowing what a form guide is, where to find it, how to read and interpret it, where to place a bet, and emotional and affective responses to winning (such as joy or excitement) or losing the bet (such as disappointment or anger). In a sense, the body and mind are work together to help people perform social practices.

Carrying out a practice also normally requires the use of materials—any objects or things that are needed to perform them. These materials are an element that serve as indispensable resources needed to perform any given social practice [47]. In our above example, the form guide usually found in a newspaper is required, as is a pen to fill in the betting slip, the betting slip itself is another material, and the betting shop and the building in which it is located is another set of materials. Language and discourse are also an element of practice serve to influence how they are performed. Discourse can be explained as systems of thought comprising ideas, attitudes, actions, and beliefs that serve to construct social subjects and the social worlds that they relate to [56]. In practice theory, this can involve the use of certain words, terminology, phrases of forms of speech to talk about, discuss and frame the understanding of a social practice. In our example of betting on a horse race, language and discourse will often frame understanding of the conditions of the race course, the form of the runners and riders, the value in the odds on certain horses, tips on which horse might offer a good bet, and even how the placing of a bet is described—for example whether an each way bet or a bet to win on the nose is made.

Social norms, which can be explained as informal and unwritten rules that govern the behaviour of people, are an element that can also shape practices. Such social norms can influence whether the practice is performed in the first place, and how the practice is performed. So, the desire to place a bet on a horse race may be shaped by social norms—for example when the Melbourne Cup in Australia or the Grand National in the UK is held, it is a normative expectation that people will place a bet on the race. Once the desire to place a bet has been realised, there are unwritten rules about how this is done, and how one should behave when placing a bet. Spaces and places are elements that may also affect how a given social practice is performed. For example, the desire to place a bet on a horse may be increased by being in a betting shop, or a friend’s house in which the horse racing is being shown on Television. The social structures in which a gambler finds themselves may also influence betting practices. As an example, if family members, friends, or acquaintances are present and are also placing a bet then this may influence a person to place their own bet on the race. In the gambling context, social structures may also relate to the market and therefore in liberalised gambling markets, inducements to betting may be higher than under other social jurisdictions.

Furthermore, power which refers to the ability to influence or control the behaviour of people, and agency which refers to capacity of individuals to act independently and make their own free choices, are important elements of practice. For example, a person who has previously engaged in problem gambling may be tempted to place a bet on the Melbourne Cup and encouraged by a peer who is a dominant individual holding power and influence in a social group. However, that person may employ their human agency to resist this temptation and decide to not place a bet. Finally, similar to other social science perspectives such as assemblage thinking [57], practice theorists argue that all of these various elements of practices are active, have agency, and therefore can affect the performance of practices and related social outcomes in different ways. As such practice theory assigns distributed agency among the elements of practice [42].

Practice theory helps supplement approaches that focus solely on human behaviour as understood through individual psychology perspectives, or macro-level analyses that focus only on social structures and their impact [45, 58]. Instead, the approach being proposed here acknowledges both structure and agency but giving primacy to neither. Instead, the focus is on how practices are performed, and what elements constitute their performance. It follows that these everyday practices are grounded upon, and embody in themselves, the ideological, temporal, spatial, and social orderings of the system [44]. At the same time, as individual actors performing social practices, they also reproduce and shape the larger organising structures and system of society. From this perspective then, gambling can be considered not simply as an aspect of a gamblers’ individual personality, attitudes, values, or beliefs—but also as part of the wider socio-cultural, economic, and regulatory structure that shapes and influences those attitudes and beliefs in the first place. As such, the performance of the social practices of gambling comes to be seen as something that is normal and routinised, embedded in the everyday rituals of life. These practices are shaped by environment and culture and yet also reinforce cultures of gambling.

Furthermore, in this tradition, researchers argue against considering gambling practices in isolation, but also urge consideration of ‘bundles’ of practices that are often woven together in everyday life to form a practice nexus [48]. Often these practice bundles form temporal sequences performed at specific times of the day, or days of week. For example, the morning routine of waking up, getting out of bed, showering, dressing, and eating is an example of one nexus of social practices. Or in relation to the gambling, this may occur concomitantly with practices of socialising, drinking alcohol, watching sport and so on. Also, social practices can occur in social contexts in which other bundles of practices take place. Using the example of gambling as a social practice, this may be related to practices involving work (e.g. running a sweep for a big horse race), holidaying (e.g. gambling in a Casino on a trip to Vegas), socialisation (betting on a sporting event attended with friends), or relaxing (gambling on the outcome of an event watched on TV at home).

Practice theorists have also drawn attention to how five various forces can frame a nexus of social practices [59]. First, attention is drawn to how certain phenomena such as affect (e.g. pleasure, anxiety, anger), general understandings (e.g. understandings of family or work), and ideology (e.g. political economy, market ideology and neoliberalism) can suffuse a nexus of practices. For instance, a person experiencing anxiety is likely to perform various social practices like working and socialising through an anxious disposition. And it could be argued that the march of neoliberalism, concomitant with globalisation, marketisation, and liberalisation, has suffused the nexus of practices that form the global gambling industry [12].

Second, certain objects or a specific practice can thread through a nexus of practices—for example caring for children may frame how various practices like cooking, cleaning, and washing may occur in a family household. Third, the idea of largeness refers to how various social practices connect and form practice complexes from small (e.g. practices in the family household) to large (e.g. markets or international relations). Fourth, practice nexuses are subject to changing connections and shift and change in various ways over time, space, jurisdiction, and materiality. For example, as a person ages, has a family, moves location, or lives under different political and regulatory jurisdictions, the various practices they perform will be altered. Fifth and finally, people act as practitioners of social practices and nexuses of practices and perpetuate and transform them through their actions [59].

Social practice ideas are now being used to research, understand, and inform policy and programmes regarding issues like public health and wellbeing [60, 61], alcohol consumption [62, 63], smoking [64], cycling [65], and energy consumption [66]. Researchers working in these areas have identified how practice theory can offer policymakers more nuanced understandings of social issues and direct policy in areas such as sustainable consumption, energy efficiency, obesity, and alcohol and tobacco consumption [63, 64, 67, 68]. Furthermore, government and policymakers are beginning to engage with practice theory with submissions made to parliamentary enquiries [69], and the UK Department for Environment, Food and Rural Affairs and the Scottish Government now co-funding research initiatives and developing policy initiatives based on these ideas [70, 71]. Recently, researchers have identified the potential of practice theory for gambling research [63, 72]. Embracing this potential, we set out some ideas for how practice theory and understanding gambling as a social practice can help inform the gambling research.

## How can social practice theory inform gambling research?

A social practice perspective on gambling can encourage us to acknowledge that often ‘consumption occurs within and for the sake of practices. Items consumed are put to use in the course of engaging in particular practices’ ([50], p. 145). Practice theory can help foster a shift in gambling research from a focus on gambling as related to individual choice, or as entirely configured by political, economic, and social structures. This is possible because practice theory provides a way to acknowledge both structure and agency in gambling in which there is an acknowledgement of the body [51], mental activities, discourses [46], materials [73], social norms [47], and social structures [49, 58]. These ideas are particularly relevant given the fast-changing gambling environment in which the political economy through neoliberalism, globalisation, liberalisation, markets and marketing, product and services, technologies, and social contexts of gambling is shifting [12, 74].

Taking a practice theory perspective on gambling enables us to consider it not as a discrete behaviour, but to acknowledge gambling as a social practice. Social practices of gambling may draw upon multiple elements such as bodies, discourses, norms, and materials in their performance, and gamblers themselves could be recognised as the carriers of practice. Furthermore, social practices of gambling may be bundled with other practices of everyday life such as drinking alcohol, socialising with friends, or watching sports. When we think about gambling, there is often a multiplicity of practicalities involved. People often have their own different ways of doing things, for example gambling alone at home, or in the pub with friends. Also, there are socio-cultural narratives around how gambling can help foster mateship and togetherness, or discourses regarding competencies on how to gamble, estimating the odds are, game playing strategy, and how to respond to winning and losing [20].

The body is also used, a person may use their arms, hands, and eyes to read a form guide, or press the buttons on a fruit machine or mobile phone sports betting app. Materials are used such as the mobile phone on which the app is loaded, newspapers and form guides to particular sports on which one may bet, and machines such as FOBTs, and physical buildings such as casinos. Social structures and agency also come into play—for example market regulations often dictate how, where, and when people can gamble, and gamblers may use their agency in social situations to influence the betting decisions of friends. And as mentioned earlier, social practices of gambling are often bundled together with other social practices such as socialising, drinking, and enjoying sport.

A key question here then is how can social practice theory perspectives inform gambling harm reduction initiatives? Existing gambling interventions often target individuals and their gambling behaviours [16], not social practices of gambling, how they are performed, what elements of practice are used to perform them, and how practices of gambling are carried and shaped. Gambling is commonly framed as a singular concept of harmful behaviour by ‘problem’ individuals, subject to universal laws, resulting in standard methods for harm reduction research, policy, and practice that pays little attention to the potentially important differences in how various gambling practices may be configured, and how and why they might vary. Therefore, we argue that gambling harm reduction policy should reframe the central issue not as changing individual gambler behaviour, but as changing wider practices of gambling. This would require redefining the research field and understanding that gambling is not simply caused by the personalities, values, beliefs, and choices of gamblers. Instead, it would encourage us to focus on how practices of gambling develop, and how harm reduction policy and practice could be used to reconfigure the landscape in which gambling practices do or do not take hold, see [75].

A strategic and holistic harm reduction strategy for gambling using the lens of social practice perspective may instead focus on acknowledging and altering the availability, form, and interactions between all the elements of gambling practice. For example, policies and programmes could seek to influence how the body shapes gambling practices, by regulating how gambling marketing and gambling products use design, visuals, colouring, and messaging to appeal to the senses. Policies and programmes that seek to work against the mental activities employed to routinely perform gambling practices by instead providing gamblers with the mental acuity to resist the urge to gamble in a problematic way, foregrounding the possible negative emotional and affective responses to problem gambling, and providing gamblers with other sources of emotional fulfilment through alternative leisure pursuits offers another possibility. Harm reduction strategies could also seek to limit access to materials necessary to gamble such as restrictions on EGMs and/or FOBTs, regulating the promotion and use mobile phone betting apps, or account locks on such apps for problem gamblers.

Policy restrictions on the spaces and places in which gambling may or can occur, and public discourse and media campaigns that seek to shape social norms and challenge the perception emerging in some countries that gambling is a normative practice offers another possibility. Policymakers also have the power to reshape social structures that influence gambling practices. The burgeoning gambling market found in countries such as Australia and the UK could be restricted through legislation and regulation. Finally, harm reduction approaches that seek to reduce the power and influence of the global gambling industry and equip people with agency to resist gambling could also be explored. However, such suggestions would need to account for the power and influence of the gambling industry operating as a business to generate profits, and the role of governments who collect tax revenue from gambling activity [12].

Ideally, these interventions to shape and reshape the various elements that influence gambling practices would form a coherent and holistic overall strategy to tackle gambling-related harm. Practices are often shaped by multiple forces beyond the individual such as businesses, governments, various economic and social policies, and cultural and social trends. In terms of mitigating the harms of gambling, this would necessitate a shift towards more holistic policymaking and harm reduction strategies that pay attention to these forces, and also consider how and why gambling practices interact with other practices of everyday life.

## A social practice gambling research agenda

Here we offer some propositions for what a practice theory research agenda for gambling might look like and invite other researchers to contribute to knowledge production in this area. We do not claim that our suggestions offer a definitive and exhaustive list but will help identify some key areas of focus. To help set out a research agenda, we consider some key research questions, topics and contexts, constructs of interest, and methodologies for conducting social practice research on gambling to help inform harm reduction strategies.

A key element of social practice that could inform gambling research is a focus on bodies. The concept of embodiment focuses on understanding the relationship between the body, mind, cognition, and social practices [76]. It is argued that understanding what the body is doing when people perform social practices such as eating, washing, or in the case of this article gambling, can offer deeper insights about the performance of social practices [76]. People use their bodies in various ways when gambling, for example they use their eyes to view a game or read a form guide, their arms and hands to touch a screen or pull a lever, or they may rely on nerves or a gut feeling in their stomach to place a particular bet. So, as well as focusing on how cognition shapes gambling behaviours, attention should also be drawn to how bodily movements influence gambling and may be altered to tackle problem gambling. Yet, existing gambling research says little about the place of the body in gambling and how embodiment shapes gambling practices.

Social theorists have used embodiment perspectives to understand the social world that could inform gambling research. For example, Pierre Bourdieu’s concept of habitus studies embodied dispositions such as posture, accent, and the tendency of people to hold and use their bodies in certain ways, and how these are shaped by culture and history, and as a result shape social action [41]. As an example, body language and knowing looks among a group of gamblers could act to influence how members of the group act [20]. Gambling research on embodiment could therefore consider research questions on how bodies and mental activities influence the ways that gambling practices are performed, and what role the body plays in driving harmful gambling outcomes.

Another important element of gambling practice that can form the focus for research is materiality. Gambling relies on the use of a range of materials, objects, and technologies such as mobile phones, apps, machines, cards, technologies, and rules and guides. Social practice perspectives draw attention to the role and importance of materials in the performance of practices such as gambling. In effect, practice theory attaches agency and meaning to materials as they help shape practices. Gambling research could focus on mapping what, how, and why materials are used during the performance of gambling practices, and whether the removal of important materials can work to trammel harmful gambling.

Furthermore, language and discourse as elements of gambling practice offers warrant attention. For instance, researchers have highlighted how government and industry discourses frame gambling as a positive contributor to society, and problem gambling as an individual responsibility of addicted gamblers [16]. Extant research has also demonstrated how the gambling industry uses language relating to humour, mateship, success, and sexuality to shape social discourse [32]. However, discourse and language are also used to discuss specific gambling practices and the elements of these practices, for example sports betting may include discussions on how a game attended went or speculating on the outcome of a forthcoming match. Further research on the language and discourse used by gamblers themselves could help add to the knowledge base here. Therefore, work that builds upon prior research can question how the discourses of gamblers themselves, as well as societal discourses, frame gambling practices.

Social practice theory research could also develop increased understanding of how social structures, and power and agency influence gambling. Such work would consider issues such as how do the dynamics between people and their social environment shape gambling practices. Existing gambling research has suggested that social group dynamics can have a major influence on gambling practices with power hierarchies among friendship groups shaping expectations on how to bet, how much to bet, and even how to spend winnings [20]. As some forms of gambling such as mobile sports betting become established as normative social behaviours, future research could consider questions such as how opinion leaders and influences may use their agency to shape the gambling practices of others within specific social groups.

A practice theory agenda for gambling research could also focus on questions about how do spaces and places shape gambling practices. Paying attention to the spaces and places of gambling is an idea that draws on well-established knowledge from the social sciences that space matters and plays a crucial role in shaping the social world and influencing social outcomes [77, 78]. Space is not something that is fixed and universal. Indeed Doreen Massey [77] argues that places do not have single but multiple identities, they are not frozen in time but are dynamic, and places are not enclosures with a fixed inside and outside. As an example, the emergence of mobile phone sports betting over the past 10 years has led to significant changes in how gambling practices are performed [79]. Sports betting using mobile phone apps can now be carried out in virtually any space and place in which there is access to the web through Wi-Fi or a mobile phone data network. Therefore, the spatial element of gambling, and particularly mobile sports betting could form an interesting focus for research. Currently, little is known about how the spatiality of mobile phone sports betting influences gambling practices and gambling-related harms. Specific research questions that may be considered here include, does the ability to bet virtually anywhere led to increased risk and harms to gamblers, families, and the community (see [20]). Or, research could ask—how does gambling occur in certain spaces and not others, and why (see [80]).

Building upon the idea of how spaces and places influence sports betting practices, research in this area could also draw upon a mobilities perspective [81]. Mobilities is a research paradigm in the social sciences that explores the movement of people, ideas, and things and considers the broader social implications from this [82]. Mobilities research on other social practices such as driving, food consumption, and health has offered new insights on how the movement of bodies, materials, and communication shapes social outcomes [83, 84]. Applying this thinking to gambling, sports betting can be practiced in multiple spaces and places and these are often not fixed. Gambling is not simply tied to once specific space and place. For example, young people on a night out may transition from a sports event, to a pub, to a friend’s house whilst betting on sports. So, gambling research using these ideas could focus on questions such as how do gambling practices play out over the course of a day, or an evening.

Importantly, practice theory work that considers these various elements of gambling practice should seek to explore how the different elements of practice work together to shape outcomes. Therefore, practice theory work on gambling would not necessarily focus only on bodies, or materials, or language and discourses. Rather, practice theory research in the field should seek to examine how different elements are enlisted and come together to perform gambling practices, and also pay attention to the relational dynamics between these elements of practice. This is important, as it can help also inform how holistic harm reduction strategies may seek to work at multiple points of intervention by simultaneously tackling how bodies, materials, norms, discourses, and social structures shape gambling.

Finally, a future research agenda could explore research questions that focus on how do gambling practices connect to form a nexus practices with other social practices such as alcohol consumption or socialising with friends? Practice theorists refer to bundles of practice [48] recognising that practices are rarely performed in isolation but as part of a nexus of practices, or constellations of events [59]. Gambling often coincides with other practices such as eating and drinking, consuming sport, and socialising among friends [85, 86]. Future research could focus on the nexus of gambling with other social practices like alcohol consumption and the consumption of sport and how this shapes behaviours and outcomes. Furthermore, harm reduction strategies could consider not only a specific focus on harmful gambling practices, but practices performed alongside gambling such as alcohol consumption.

Taking a nexus of practices, perspective also offers a framework for understanding how affective forces, the political economy, or general understandings may suffuse such practice bundles that involve gambling. Such work could for example consider how neoliberal ideology, globalisation, and marketisation suffuse practices of gambling in the emerging economies of Africa, through alignment with ideas of status, success, and Western modernity. A nexus of gambling practices approach could also consider how certain practices such as a regular social gathering among friends may thread through the nexus and incorporate routines of gambling, drinking, socialising and so on. Longitudinal research that considers changing connections between gambling and other social practices through time, space, jurisdiction, and materiality can also help develop understanding.

Finally, an important consideration for any social practice research agenda for gambling is what methodologies can be used for inquiry. Given the broad focus of social practice theory on elements of and the performance of practice, interdisciplinary research drawing upon multiple methods is often used. Traditional qualitative methods such as focus groups, and narrative interviews have been used to offer insights about the performance of gambling practices [20, 72]. However, more novel and innovative methods are often used in social practice theory research. Methods such as ethnography and visual ethnography can be useful for capturing how language, materials, and bodies are used in the performance of practices [87]. Ethnographic and visual ethnographic methods offer possibilities for the researcher to pay attention to the fleeting, unspoken, mundane, ongoing, eventful, and happenstance of everyday life. Observation research of people performing gambling practices, or video ethnographies that capture people, bodies, spaces and places, language, and social interaction during the performance of gambling practices could add considerable insight to our understanding of what may drive gambling-related harms. Gambling research using such methods would need to be conducted in an ethical manner, in which trust, consent, and decisions over the use of visual data are carefully considered.

Cognitive neuroscience has also been suggested as a way to look at social practices and specifically how mental activities such as emotion and cognition influence their performance, or even how the eyes are used and where they focus during events [88]. Cognitive neuroscience methods can provide useful insights into how external stimulus such as people, events, materials, and time affects people’s information processing and decision-making in the brain [89]. In the context of gambling, interdisciplinary research could combine electroencephalogram (EEG) or functional magnetic resonance imaging (fMRI) studies that measure how people’s brains respond to gambling stimulus such as sports betting apps, with complementary ethnographic work observing how people use such apps. Such interdisciplinary research between social scientists and neuroscientists is being carried out in other areas such as energy conservation behaviours [89].

Similarly, neuroscience methods could measure brain responses to the use of FOBT machines, or eye tracking research could consider what people are looking at and how they react to gambling advertisements in combination with qualitative narrative interviews to give insights on how the mind and mental activities may shape gambling practices. Such research approaches would also need to consider important ethical questions relating to neuroscientific research [88] such as potential effects on participants from exposure to gambling stimulus, and provide relevant support services as necessary. Finally, Meier et al. [63] suggest that quantitative approaches to social practice research that quantify various elements of practice that shape a potentially harmful practice such as gambling could add new understanding. Indeed, some quantitative studies in alcohol research record information that offers insights about the elements of practices of alcohol consumption such as location, social context (e.g. presence of family, colleagues, friends), and day and time [90]. As Meier et al. [63] argue such approaches could be applied to gambling research as well. The use of innovative research methods to investigate gambling as a social practice holds potential to inform more nuanced gambling harm reduction policy and programmes as well as provide stimulus for future research in the field.

## Conclusions

This paper argues the case for a practice theory turn in the gambling research field. We argue that such an approach is warranted given the dynamic and multifaceted nature of gambling and the considerable health and social harms caused by gambling. Furthermore, our clear identification that gambling practices are performed using multiple elements of practice such as bodies, materials, spaces and places, and language and discourse support our case for a turn to practice. We have argued that practice theory research can add to the nascent stream of socio-cultural research on gambling [14, 15, 17–19, 23, 24] to provide nuanced and alternative understandings that can complement existing individual psychology of behaviour perspectives, regarding the personal, social, and cultural dimensions that shape gambling. In this article, we have outlined what practice theory is, how it is relevant to gambling, and can help strengthen the research evidence base and suggested some ideas for a future research agenda in this space. We call upon social science researchers from across disciplines to embrace the challenge and engage in practice theory gambling research to complement existing knowledge, add to our current understanding, and help inform more effective gambling harm reduction activity into the future.

## Data Availability

Not applicable
